# Robust parameter extraction for decision support using multimodal intensive care data

**DOI:** 10.1098/rsta.2008.0157

**Published:** 2008-10-20

**Authors:** G.D. Clifford, W.J. Long, G.B. Moody, P. Szolovits

**Affiliations:** 1 Harvard-MIT Division of Health Sciences and Technology, Massachusetts Institute of TechnologyCambridge, MA 02139, USA; 2 Computer Science and Artificial Intelligence Laboratory, Massachusetts Institute of TechnologyCambridge, MA 02139, USA

**Keywords:** clinical errors, data fusion, decision support, intensive care unit, noise, signal quality

## Abstract

Digital information flow within the intensive care unit (ICU) continues to grow, with advances in technology and computational biology. Recent developments in the integration and archiving of these data have resulted in new opportunities for data analysis and clinical feedback. New problems associated with ICU databases have also arisen. ICU data are high-dimensional, often sparse, asynchronous and irregularly sampled, as well as being non-stationary, noisy and subject to frequent exogenous perturbations by clinical staff. Relationships between different physiological parameters are usually nonlinear (except within restricted ranges), and the equipment used to measure the observables is often inherently error-prone and biased. The prior probabilities associated with an individual's genetics, pre-existing conditions, lifestyle and ongoing medical treatment all affect prediction and classification accuracy. In this paper, we describe some of the key problems and associated methods that hold promise for robust parameter extraction and data fusion for use in clinical decision support in the ICU.

## 1. Introduction

Intensive care provides one of the most challenging locales for both clinicians and engineers who try to support clinical activities. Intensive care unit (ICU) patients are often the sickest, frequently having several pathophysiological problems that must be managed simultaneously to avoid death or severe morbidity. Both physiological state and external interventions change frequently, demanding rapid analysis and quick, high-stakes decisions.

Advances in the development of technology, computational signal processing and biological modelling have led to a growing interest in the archiving and use of extensive hospital–medical databases. Although current clinical practice is centred on human expert assessment of the correlations between parameter values and symptoms, there is a growing awareness within medical communities that the enormous quantity and variety of data available cannot be effectively assimilated and processed without automated or semi-automated assistance. Automated systems have been in place in the ICU and the operating theatre (OR) for several decades, including automated arrhythmia analysis of the bedside electrocardiogram (ECG) and low (or high) oxygen saturation warnings from the photoplethysmograph (PPG). However, each device acts in an isolated fashion with no reference to related signals or an individual's prior medical information, such as genetics or medical history. Some patient-specific data are used in an ad hoc manner, such as body weight (for the adjustment of drug rates), allergies (to restrict medications) and age (for arrhythmia alarm thresholds). However, automated use of such information is rare.

In this paper, we outline a long-term approach to develop systematic bases for extracting information in order to provide assistance to clinicians faced with the enormous challenges of providing high-quality ICU care. We are currently collecting large datasets of actual patient experiences in the ICU, developing methods to analyse and abstract those data, retrieval systems to allow selection of events of interest, creating models that relate such data to patients' clinical conditions and pathophysiological status, and building both physiological and statistical models to enable sophisticated decision support systems that base alarms on an integrated view of the patient and that can assess or even suggest alternative courses of action. Although we frame these ideas within a wider context, here, we focus principally on the problems we have encountered and the solutions we have developed for collecting ICU patient data and extracting information that is useful for decision support.

We have instituted a large-scale systematic collection of data about ICU patients (Saeed *et al*. 2002) to provide a baseline understanding of what currently happens in the ICU and to allow us to learn to model patients' conditions and their responses to various interventions. These data derive from a heterogeneous set of sources, including bedside monitoring equipment; clinical observations by doctors and nurses; laboratory measurements; records of both continuous and discrete drug administration; reports from physical examinations, referring physicians, radiologists, pathologists and other specialists; and records of past conditions, treatments and outcomes, as normally recorded in discharge summaries. Each of these sources of data carries its own set of technical problems, ranging from mundane issues of data standardization to difficulties in assessing the quality of recorded data and dealing with missing data. To date, we have collected data on approximately 30 000 patients from four different ICUs at a large tertiary-care teaching hospital. Approximately 10 per cent of those records include high-frequency physiological signals recorded from the bedside monitors, including multichannel ECG, invasive arterial blood pressure (ABP) and PPG. Associated derived parameters, such as heart rate, systolic/diastolic blood pressure and oxygen saturation, are also available.

In the following sections, we describe issues we have encountered in

- the collection, measurement, transmission, transcription and storage of these data,
- abstraction and robust parameter extraction from often noisy and incomplete data, and
- extraction of clinically relevant concepts from unstructured text, which is the form in which many notes and reports are stored.

## 2. Errors in collection, measurement, transmission, transcription and storage of data

The first problem faced in gathering ICU data is the necessity of collecting the data from a variety of sources: instruments that acquire and digitize continuous signals; devices such as respirators and intravenous drug delivery systems that must be interrogated to record their settings; laboratory results that may have been automatically or manually transcribed into clinical information systems; and medical histories, admitting notes, progress notes, problem lists and discharge summaries that must be obtained from online or paper medical records. These diverse data streams must be synchronized in order to make sense of their inter-relationships. Even establishing basic causal relationships among data elements (did the patient's symptoms prompt a change in medication, or did the medication change cause the symptoms?) can be problematic when time is recorded by independent and unsynchronized clocks. At an even more basic level, it is necessary to match each recorded data stream with the correct patient among all those currently in the hospital, a problem that should be trivially easy to solve but is often complicated by instruments with poorly designed set-up procedures that are not always completed in the context of urgent care, human transcription errors (Hug & Clifford 2007; Vawdrey *et al*. 2007) and policies intended to protect patient privacy. The use of proprietary formats and protocols for medical data storage and communications is slowly giving way to open formats (e.g. EDF, WFDB, XML; Clifford *et al*. 2008) and protocols (such as HL7 and the IEEE medical information bus standard P1073; Alsafadi *et al*. 1994), which will reduce transcription errors. However, the processes of capturing and digesting these data into a consistent format remain complex.

Having solved all of these problems by whatever means necessary, it may be possible to assemble an electronic medical record containing most of the information upon which medical decisions are founded. Just as traditional medical records may contain errors from a variety of sources, so may their electronic equivalents. Measurement errors may stem from incorrect calibration, improperly located or malfunctioning transducers, artefact, environmental noise, or from errors in transmission, transcription, storage or retrieval (Clifford & Oefinger 2006). Some of these errors can be minimized by use of good biomedical, computer and human engineering practices, for example by stabilizing sensors to limit motion-related artefact; minimizing the area of low-voltage sensing circuits and using shielded cables to avoid signal contamination by induced currents; using error detecting and correcting codes when transmitting and storing packets of digital data; employing data communication protocols that incorporate handshaking, retransmission and redundancy to avoid data loss; redesigning workflow to capture data automatically, to avoid manual transcription of data where possible, and to verify data that must be transcribed at the time of transcription; and regular reviews of data collection practices to identify and address deficiencies.

Conscientious use of best practices can reduce but not eliminate measurement errors. The effects of those errors that remain upon subsequent analysis of the record can be minimized by searching for logical inconsistencies (such as when the systolic blood pressure is lower than the mean or diastolic blood pressure), or by comparing multiple redundant measures of the same physiological parameters. For example, heart rate may be derived from the ECG, an invasive ABP line and the PPG. These issues are explored further in §3*d*, after discussing problems and solutions related to noise reduction, signal quality, artefacts and missing data.

## 3. Abstraction and robust parameter extraction

In order to provide information for medical experts (or automated decision support systems) to make choices concerning patient care, the wealth of available data must be reduced to a set of distinct concepts and features. Although many parameters are derived from patient data ‘on the fly’ and recorded for later review, trust metrics or signal quality measures associated with these parameters are rarely stored. Therefore, it is difficult to ascertain the credibility of a given parameter unless the original data from which the parameter was derived are available, either to visually verify the data or in order to derive independent quality metrics.

Noise reduction algorithms often introduce misleading distortions in medical time-series data and, therefore, they should be applied only when the data are determined to be too noisy for a feature extraction algorithm to be applied accurately. However, it is often necessary to extract features and compare them with a population norm, or a patient's history, in order to determine whether significant amounts of noise are present. A method for simultaneously (or recursively) extracting features and estimating noise levels is, therefore, appropriate.

In this section, we describe a generic approach to noise reduction and signal quality analysis, together with a data fusion framework that provides for a robust estimate of extracted physiological parameters that evolve over time.

### (a) Noise reduction

After appropriate formatting, storage and initial coding of data, perhaps the most important stage in data processing is the application of signal processing algorithms to deal with the noisy and transient nature of physiological signals. Even when the data are continuously available and the sampling rate is high enough, data can still be masked by periods of intense noise due to movement artefact. Strategies for detecting and (where possible) removing noise in physiological data depend on the nature of both the noise and the data source and typically include infinite impulse response filters, finite impulse response filters, principal component analysis (PCA; Moody & Mark 1989), independent component analysis (ICA; McSharry & Clifford 2004; He *et al*. 2006) and wavelets (Addison 2005). Much of the data recorded in the ICU are nonlinear or non-stationary, however, and the mixing between the noise and the signal is also non-stationary. Therefore, techniques such as ICA cannot work reliably unless calculated over small, quasi-stationary segments of data and frequently updated. Furthermore, the noise and the signal are not independent, such as when heart rate increases due to activity are associated with increasing frequency of artefact (Clifford *et al*. 2002) and methods to separate them, which assume independence (such as PCA and ICA), only work when the coupling between the signal and the noise is weak. It should be noted that PCA-based techniques have proved extremely effective for filtering on a beat-by-beat basis, particularly in applications such as QT analysis (Okin *et al*. 2002), ST analysis (Moody & Jager 2003; Jager *et al*. 2004), QRS subtraction and QRS classification (Moody & Mark 1989).

Takla *et al*. (2006) provided a thorough review of the types of contamination of signals in the OR and methods that have been proposed to deal with the noise. Although much of this information is pertinent to monitoring in general, the ICU is more problematic than the OR, since the latter is more highly controlled, with a higher staff-to-patient ratio. ICU data are often only available on an infrequent basis (relative to the underlying dynamics), and removal of noise becomes problematic. The best method to deal with noise is often simply the use of a median filter to reject outliers (Mäkivirta *et al*. 1991). For example, heart rate and blood pressure averages recorded by nursing staff every hour sometimes exhibit artefacts that are significantly different from the underlying waveform data (Hug & Clifford 2007). Although a median filter is able to reduce the average magnitude of the error in this scenario, this is only because patients tend to be stable and exhibit the same physiological parameter values from hour to hour. However, there is no guarantee that the *outlier* is not a real event. In fact, it is the rare outliers that are often of interest in biomedical time-series data, since they indicate that the aim of managed stability for a patient may be unsuccessful and that changes in treatment are required.

To allow for the non-stationary nature of ICU data, an adaptive filter is often more appropriate, where the transfer function changes in response to each new data sample or feature (Takla *et al*. 2006). Adaptive filters are generally either ad hoc (Martinez *et al*. 1997; Husoy *et al*. 2002) or model-based (Clifford *et al*. 2005; Clifford 2006; Sameni *et al*. 2007). Although model-based filters provide a much more effective suppression of noise, they tend to be more computationally intensive, and their effectiveness is dependent on the accuracy and applicability of the model employed. In Sameni *et al*. (2007), the authors recently proposed an adaptation of a model-based filtering approach to the ECG, which is particularly suitable to a real-time implementation. By using an unscented Kalman filter, a nonlinear version of the Kalman filter (KF), they leveraged the beat-to-beat dynamics (and similarities) to allow a computationally efficient Bayesian approach to ECG model parameter estimation. Although Sameni *et al*. demonstrated that the technique outperforms the best of previously described ECG filters on normal sinus rhythm ECGs, performance on arrhythmic data is unclear. In all likelihood, unless a pre-classification algorithm is employed, the model will have to be refitted to the data for moderate changes in morphology, and classified as abnormal or artefactual. It is also worth noting that, since the model is based upon a superposition of Gaussians, it is easily adapted to filtering and classification of other cardiovascular signals such as the blood pressure (Clifford & McSharry 2004; Clifford *et al*. 2005).

### (b) Artefacts and missing data

Most filtering techniques are also sensitive to artefacts and missing data. In particular, even when signals have been sampled above the Nyquist limit, intervals of missing data may be frequent, due to disconnections, sensor errors, equipment changes, intrusive diagnostics and request-based data (such as blood tests). Sometimes noise and artefact can be so high that it is best just to discard the section of data, effectively making an evenly sampled signal irregularly sampled.

Often the sampling frequency is inherently uneven, particularly in the case of diagnostic data, which are ordered when an event or combination of observations indicate that a particular test is required. Missing data and irregular sampling are highly related concepts, although the former implies that useful data may exist between each sample point and may carry further information about the state of the patient (such as a significant change in a given variable). Some form of interpolation may, therefore, be useful in estimating the unobserved information. However, it may not be appropriate to guess the values of missing or hidden data, since any slight error might lead to an erroneous decision; there are cases when an estimate is useful. Furthermore, reporting the bounds of error in an estimate allows a clinician to make safe harbour decisions.

For single-parameter time series, little more than a sample-and-hold approach (with a time-out) is generally used to fill in the missing data. This is generally a good approach for frequently sampled data from ICU patients who are usually managed for stability, and thus exhibit infrequent large changes in the value of a physiological parameter. However, the situations that are often more interesting and informative are the infrequent changes and resampling schemes are often used (such as sample-and-hold, linear or cubic spline interpolation). However, these approaches introduce spurious low- and high-frequency noise and can be extremely sensitive to the number of missing data points or to the irregularity of the missing data (Clifford *et al*. 2005).

Other more complex methods for filling in missing data involve using the statistical and/or dynamic nature of the data (rather than just neighbouring gradients) to form estimates of the intervening sample values such as min–max interpolation (Fessler & Sutton 2003), autoregressive modelling (Rajan *et al*. 1997; Cassidy & Penny 2002) and KF methods (Chin 2001; Yarita *et al*. 2007). Sometimes, however, it is more appropriate to use methods specifically designed to be used with missing data (or irregularly sampled signals). For spectral estimation, the Lomb–Scargle periodogram (LSP; Lomb 1976; Scargle 1982) is a particularly robust method for extracting frequency estimates of unevenly sampled data, and has been shown to be particularly suited to spectral quantification of heart rate variability (Moody 1993; Laguna *et al*. 1998). The LSP does not require the interpolation of any data, as it performs a least-squares fit of sinusoids at each frequency to form an estimate of the power spectral density. The LSP has been shown to be relatively insensitive to the density of missing data and removed artefact with relatively insignificant changes for up to 20 per cent missing data (Clifford *et al*. 2005).

When multiple sources of related information are available, it is possible to exploit the covariance of the data, such as when using PCA or imputation. However, such techniques again assume stationarity of the dynamics of the data (unless incremental updates are calculated on a frequent basis), and they require that the missing data be missing at random or that an accurate model of how the missing data are distributed be known. Furthermore, these techniques are sensitive to outliers and non-removed errors. Since no accurate model exists of how missing or noisy data are distributed, the interpolation of missing ICU data is extremely difficult (Abdala & Saeed 2004). (In general, data are missing because they are perceived to be irrelevant for the current clinical problems, or because exogenous interventions or endogenous activity has rendered the data useless. Neither of these circumstances is random, or amenable to simple models.)

It should be noted that the frequency at which a parameter needs to be sampled depends on both the parameter type and the question we are asking about the patient. For example, although blood pressure can exhibit large changes over a period of a few seconds (e.g. during a head-up tilt), if we are looking for evidence of haemorrhage, we may not need to sample more frequently than once every 5 or 10 min to capture the dynamics of the situation. The required sampling frequency is also related to the intrinsic dynamics of the parameter, so that heart rhythm, which can change over a few beats, is sampled rapidly (at 100 Hz or more), whereas blood creatinine (abnormal levels of which indicate renal insufficiency) may change only over hours. Consequently, creatinine values are sampled much less frequently and can be reliably interpolated over several minutes, whereas heart rate estimates cannot. The effective Nyquist frequency for a particular parameter also depends on an individual's physiology and medical condition, and so it is difficult to be sure whether parameters are being undersampled. However, clinical teams tend to sample parameters more frequently when they believe a patient may be unstable with a rapidly changing (usually degenerating) physiological condition. Therefore, the clinical team often notices signs or symptoms indicative of rapid changes and adjusts the sampling rate so that loss of important information does not occur.

### (c) Signal quality analysis

Since robust methods for dealing with missing data are not always available, it is sometimes more appropriate to define a signal quality measure for a given data stream, and simply ignore the segments of data that have a signal quality below a given value. However, metrics for signal quality are both signal and application specific. For example, noise above 20 Hz, which does not distort ABP estimates, can disturb ECG peak detection algorithms and cause heart rate variability algorithms to report incorrect values, while leaving heart rate estimation algorithms unaffected. Low-frequency noise (below 1 Hz), which only disturbs subtle features in the ECG such as the QT interval or ST segment, can cause significant errors in the estimate of the blood pressure. A general treatment of signal quality measures is therefore not possible. However, signal quality indices (SQIs) can generally be constructed by thresholding on known physiological limits such as the maximum field strength for the ECG, the maximum rate of change of the blood pressure or the distribution of energy in the frequency domain. However, it is the relationship between physiological parameters that provides the greatest opportunity to construct SQIs. For example, if heart beats are detected in several ECG and/or pulsatile waveforms within an expected period of time, all signals can be considered to be of reasonable quality.

SQIs are generally calculated by bedside monitoring equipment but are rarely used by clinical teams or automated alert systems, since there is often an assumption that the monitor will provide either no information or a *best guess* of a parameter in the absence of good quality data. However, as we have already discussed, it is very difficult to make an accurate or useful guess of a missing parameter in non-stationary data, such as that found in the ICU, and a sample-and-hold approach is often used. Although this can be useful to a human attempting to observe the current state of the patient, this is an inappropriate solution for passing data to an automated or semi-automated algorithm.

With current trends towards semi-automated analysis, it is important that SQIs are available for each datum and, if possible, be calibrated to provide a known error for a given value of the SQI. In this way, another algorithm can make informed choices concerning the validity of the datum for a given application, and derived estimates can be provided with accurate error bounds. In Li *et al*. (2008), we calibrated a set of ECG signal quality metrics (based upon statistical, temporal, spectral and cross-spectral features of the ECG), so that a given value of an SQI metric was equated to known error in heart rate. A similar approach was also taken to ABP, and hence error bounds in derived estimates that rely on heart rate and blood pressure (such as the cardiac output) can easily be estimated from the standard compound error formula. Generally, data in the ICU are processed in isolation from other parameters and signal quality labels are therefore rarely constructed with reference to other signals. In our approach to SQI derivation, we have concentrated on the relationships between signals, such as the transit time between the ECG and the ABP (Zong *et al*. 2004) and the inter-ECG lead relationships (Li *et al*. 2008). By comparing related signals and thresholding these relationships on known physiological limits, it is possible to determine whether the data are logically consistent. Since it is rare that a sequence of extracted features will randomly manifest in a physiologically plausible manner, internal consistency between signals can indicate high signal quality on the contributing leads.

Frequently measured parameters (such as heart rate and blood pressure) are amenable to SQI analysis because there is usually an underlying rapidly sampled waveform from which the metrics can be derived. When the sampling rate of the data available drops to around 1 Hz or below, signal quality measures become problematic, since it is almost impossible to differentiate between a real physiological change and an artefact.

Errors in less frequently sampled clinical data (such as blood tests) are more difficult to determine for two reasons. First, the sampling rate is low compared with how rapidly a variable can change. (It should also be noted that there is often a considerable delay between the biological samples being sent for testing and the received results, and so an accurate knowledge of the time of the original sampling must be known.) Second, the relationship of a blood test to other signals is extremely complex, and testing the ‘truth’ of a measurement would require an extremely complex and accurate model of an individual's physiology. The general approach is that a clinician makes a hypothesis concerning the outcome of the test, based upon current monitored data and a medical history. If the prediction turns out to be accurate, then the belief in the result is high. Otherwise, a test may be reordered, particularly if subsequent data indicate that the test results are contradictory. This type of modelling is extremely complex and the reader is referred to Long (2001) for more details.

### (d) Robust data fusion

Exploitation of the covariance structure of the data is one method of data fusion, since a non-diagonal covariance matrix implies redundancy in the individual data streams. However, weighting different data streams can be difficult when the measurement units are different and the distributions of the data are different (and non-Gaussian). For instance, what blood pressure change (measured in mm Hg) is equivalent to a 5 per cent drop in oxygen saturation? Townsend and Tarassenko (Nairac *et al*. 1997; Tarassenko *et al*. 2001, 2002*b*) approached this problem by using a large ICU database to renormalize five clinical parameters (heart rate, blood pressure, respiration rate, oxygen saturation and temperature) to zero-mean unit variance. Large statistical deviations in this five-dimensional space equate to abnormality that has been shown to be predictive of future interventions (Tarassenko *et al*. 2006).

When the data to be fused pertain to the same parameter, the data fusion is technically more straightforward, although it requires a method for arbitrating between conflicting estimates. In a recent work, we have extended the work of Tarassenko and Townsend (Tarassenko *et al*. 2002*a*, 2003) to fuse multiple observations of different physiological signals (such as heart rate and blood pressure) from multiple sources, recorded at arbitrary times, within a KF framework (Li *et al*. 2008).

The KF is an optimal state estimation method for a stochastic signal that estimates the state of a discrete-time controlled process, *x*, with observable measurement data *z*. The KF uses the *a posteriori* state estimate ${\hat{x}}_{k}^{-}$, a state transition matrix ***H*** and the *Kalman gain* ***K***_*k*_ to recursively predict the *a priori k*th state estimate, such that

(3.1) $${\hat{x}}_{k}={\hat{x}}_{k}^{-}+K_{k}\left(z_{k}-H{\hat{x}}_{k}^{-}\right).$$

The Kalman gain is given by $K_{k}=P_{k}^{-}H^{\text{T}}{\left(HP_{k}^{-}H^{\text{T}}+R\right)}^{-1}$, where $P_{k}^{-}$ is the error covariance of the *a priori* estimate and ***R*** is the state noise covariance. The above estimate minimizes the measurement innovation *η* (or residual, sometimes denoted *r*) given by $\eta_{k}=z_{k}-H{\hat{x}}_{k}^{-}$ (the error between the prediction and the observation).

Note that ***K*** is inversely proportional to ***R***, the measurement noise covariance, and represents how rapidly the KF will adapt to new observations. In a recent paper (Li *et al*. 2008), we have proposed a modification to ***R*** by a multiplicative factor, such that ***R***→*γ****R***, where

(3.2) $$\gamma ={\text{e}}^{\left(s^{-1}-1\right)},$$

and *s* is a signal quality threshold raging between 0 (poor signal) and 1 (excellent signal quality) inclusively. This modification has the effect of forcing a KF tracking algorithm to trust any given observation when the SQI, *s*, is high (since as *s*→1, *γ*→1). When the SQI, *s*, is low, *γ* tends to infinity and the resultant large increase in ***R*** results in a low Kalman gain. Therefore, the KF no longer trusts the current observation to make a prediction, and relies on previous observations instead. This approach turns out to provide a low error, unbiased estimator for cardiovascular time series of heart rate and blood pressure, even in extremely high noise scenarios since noisy segments of data are automatically rejected.

This KF approach also provides a robust framework for fusing multiple observations of the same parameter from different sensors. Tarassenko and Townsend (Tarassenko *et al*. 2002*a*, 2003) proposed weighting each observation, *x*_*k*_, of a physiological parameter by the inverse of the normalized innovation, *η*, for each channel. In the two-channel scenario (*k*=1, 2), the weighted estimate of a parameter becomes

(3.3) $$x=\left(\frac{\eta_{2}^{2}}{\eta_{1}^{2}+\eta_{2}^{2}}\right)x_{1}+\left(\frac{\eta_{1}^{2}}{\eta_{1}^{2}+\eta_{2}^{2}}\right)x_{2}.$$

In their application, *x* was separately given as a scalar heart rate or respiration rate. Furthermore, ***H*** was assumed to be unity and so the current state is approximately the same as the last state $\left(x_{k}\approx x_{k-1}\right)$. For beat-to-beat or breath-to-breath updates, this can be considered approximately true.

Although equation (3.3) weights observations with low innovations more heavily, the higher innovation can sometimes be associated with the more accurate estimate. Therefore, we added a scaling function to the innovation, such that $\eta^{2}\to \eta^{2}s^{-2}$, for each channel and low-quality estimates are ‘unweighted’. For *N*-channels, this becomes

(3.4) $$x=\sum_{k=1}^{N}\left(\frac{\prod_{i=1,i\neq k}^{N}{\left(\frac{\eta_{i}}{\lambda_{i}s_{i}}\right)}^{2}}{\sum_{i=1}^{N}\left(\prod_{j=1,j\neq i}^{N}{\left(\frac{\eta_{j}}{\lambda_{i}s_{j}}\right)}^{2}\right)x_{k}}\right)\;(k=1,2,\ldots ,N),$$

where the 0≤*λ*≤1 are *trust* factors for each of the channels of data. This formulation is particularly useful for the ICU data where multiple estimates of the same physiological parameter can be derived. For example, one might use the PPG, or pulmonary arterial pressure, as well as the ABP and ECG to determine physiological parameters such as HR, ABP or cardiac output.

The trust factor *λ* can be useful when two measurements of the same variable come from devices that are known to have independent and different error profiles, such as the invasive and non-invasive cuff measurement of blood pressure. In this case, the *λ* for the invasive arterial line could be set to 0.9 (to reflect a 10% error) and the *λ* for the sphygmomanometer cuff measurement could be set to 0.8 (to reflect an inherent 20% error in the reading).

This approach can also be thought of as a robust weighted interpolation scheme, with a sampling frequency of the combined set of observations. That is, the KF is updated at every observation, and every channel of data provides an estimate of the physiological parameter at a different time point. Therefore, the resultant time series has a sample point at each observation that is fit to a weighted sum of previous and current measurements.

The signal quality-modified KF approach described above involves a scalar observation model with the simplest dynamical approach (assuming that the next observation will be approx. equal to the last observation). Extensions that employ models of the dynamic evolution of the cardiovascular system, or vector KF formulations that employ models of how each signal is related to each of the other recorded signals, are likely to improve this method of tracking and noise rejection approach.

For example, Pueyo *et al*. (2008) used a KF to fuse information from the QT and RR intervals to dynamically characterize beat-to-beat adaptation of the repolarization period to changes in heart rate. Our group has also made significant progress in building both statistical (Roberts *et al*. 2006) and explicit cardiovascular models (Parlikar *et al*. 2006, 2007) for the ICU data. In particular, cycle-averaged models of blood pressure changes have proved accurate for modelling changes in the blood pressure and estimating cardiac output (Parlikar *et al*. 2007).

However, considerable barriers remain, including modelling non-stationarities in the parameters and dealing with the underlying noise. Without good methods for rejecting (or unweighting) noise, no system of modelling, data fusion or missing information estimation is likely to work reliably. Signal quality measures should therefore be evaluated on large databases, and then calculated and stored for all possible signals in the ICU.

### (e) False alarms in the ICU

One example of where we have applied the concepts of signal quality and data fusion is in the arena of false alarm (FA) suppression in the ICU. FAs in the ICU can lead to a disruption of care, impacting both the patient and the clinical staff. The resultant excessive noise pollution, desensitization to warnings and slowing of response times (Chambrin 2001) can lead to missed alarms, decreased quality of care (Donchin & Jacob 2002; Imhoff & Kuhls 2006), sleep problems (Meyer *et al*. 1994; Parthasarathy & Tobin 2004), stress for both patients and staff (Baker 1992; Novaes *et al*. 1997), depressed immune systems and longer patient stays (Hagerman *et al*. 2005).

Tsien & Fackler (1997) conducted a prospective, observational study in a multidisciplinary ICU to record the occurrence rate, cause and appropriateness of all alarms from tracked monitors. After 298 monitored hours, 86 per cent of a total of 2942 alarms were found to be false-positive alarms, while an additional 6 per cent were classified as clinically irrelevant true alarms (TAs). Only 8 per cent of all alarms tracked during the study period were determined to be TAs with a clinical significance associated with them.

Recently, Zhang *et al*. (2007) designed a system to simultaneously collect physiological data and clinical annotations at the ICU bedside, and to develop alarm algorithms in real time based on patient-specific data collected while using the system. After deployment of a prototype in a paediatric ICU equipped with a newer generation bedside monitoring system, a dataset of 196 hours of vital sign measurements at 1 Hz together with associated alarms was collected. Approximately 89 per cent of the recorded alarms were found to be clinically relevant true positives, 6 per cent were true positives without clinical relevance and 5 per cent were false positives (Zhang *et al*. 2007). Real-time machine learning showed improved performance over time and generated alarm algorithms that outperformed the previous generation of bedside monitors and came close in performance to the latest generation of bedside monitor alarm algorithms (Zhang 2007). Interestingly, this work shows that an algorithm trained only on data from a specific patient can approach the level of performance of commercial algorithms that are trained on much larger datasets (Zhang & Szolovits 2008).

Our recent analysis concerned the suppression of false life-threatening arrhythmia alarms issued by the bedside ECG monitor. Using two independent reviewers, we annotated 5386 alarms from a total of 447 adult patient records spanning 41 301 hours of simultaneously acquired ECG and ABP. A third reviewer then checked each alarm to adjudicate discrepancies and check the overall quality of the alarms. The critical arrhythmia alarm types were selected to be (i) asystole, (ii) extreme bradycardia, (iii) extreme tachycardia, (iv) ventricular tachycardia (VTach), and (v) ventricular fibrillation. Annotation revealed the FA rates of these five alarm types to be 90.7, 29.3, 23.1, 46.6 and 79.6 per cent, respectively, with an average FA rate of 42.7 per cent. An algorithm to suppress these FAs was then developed, which used a signal quality measure, *s*_*N*_, derived from the ABP waveform to decide on the truth of the ECG arrhythmia alarm. (In this application, *s*_*N*_ was actually a signal ‘normality’ index (Sun *et al*. 2006). Signal normality equates to a high signal quality and no features indicative of a non-sinus rhythm.) At each ECG alarm point, a reference was made back to a 20 s synchronous segment of the ABP waveform, and if *s*_*N*_ was higher than a given threshold, the blood pressure was considered to be commensurate with a sinus rhythm, and the ECG alarm was suppressed if the ABP-derived heart rate was too slow (or fast). The threshold, *s*_*N*_, was expected to differ for each alarm type since abnormalities in the ECG will differ depending on rhythm and heart rate. Therefore, the annotated alarms were divided into two sets: a training set and a testing set. Each *s*_*N*_ was then optimized (together with other alarm-specific thresholds, such as the number of beats from which to calculate the heart rate), to determine the highest FA reduction rate, with the lowest TA suppression rate.

This approach provided an overall FA reduction rate for the five alarm categories above of (i) 93.5 per cent, (ii) 81.0 per cent, (iii) 63.7 per cent, (iv) 33.0 per cent, and (v) 58.2 per cent, with an overall suppression rate of 59.7 per cent. This equates to an equivalent FA rate of (i) 5.5 per cent, (ii) 5.5 per cent, (iii) 8.4 per cent, (iv) 30.8 per cent, and (v) 33.1 per cent, with an overall FA rate of 17.2 per cent. However, it should be noted that invasive arterial lines are not available for all patients in the ICU (only approx. two-thirds of the population), and so to provide this level of FA suppression for all life-threatening alarms would require an extension of the algorithm to use the oxygen saturation waveform. TA suppression rates were all zero except for VTach, indicating that VTach does not always manifest as an abnormal ABP waveform, and referencing back to the ECG is required. A full description of the method and results can be found in Clifford *et al*. (2006) and Aboukhalil *et al*. (2008).

Significant work still remains in the arena of FA reduction, particularly with respect to lower priority alarms, which, although less important, still add significantly to the problem of FA pollution in the ICU. In fact, non-critical alarms constitute over 90 per cent of the alarms in the ICU. Furthermore, these alarms are not split into groups relating to clinically insignificant, clinically relevant and immediately actionable. The data and annotated alarms, a subset of the MIMIC II database, have therefore been made publicly available via PhysioNet (Goldberger *et al*. 2000; LCP 2008) in the hope that public collaboration will rapidly improve this situation.

## 4. Coding of clinically relevant events and concepts

Once parameters have been robustly extracted, they must be provided with a useful label. In the case of standard cardiovascular parameters, the label is self-evident (heart rate, blood pressure, cardiac output, etc.). However, combinations of parameters can provide a richer picture of the state or class of a patient. For example, a series of desaturations during the night followed by cessations in breathing are indicative of apnoea. The prior probability of placing the patient into a given class can be extremely important and a rich database of ICU data also provides the opportunity to extract information for such prior probabilities from alternative sources.

### (a) Medical lexicons for annotating ICU data

The objective (or semi-objective) classification of ICU data requires a standardized lexicon or system of labelling. Although such systems exist for some signals (such as the ECG), many labels rely on subjective observations with high interobserver variability. Furthermore, for many medical diagnoses, there are no agreed definitions, and the divisions between categories are fuzzy. For example, more than 30 different definitions of acute renal failure have been used in the literature (Bellomo *et al*. 2004). However, with multiple experts and a well-defined set of criteria, the labelling of a given event or condition reaches agreement levels of 95 per cent (Douglass *et al*. 2004; Neamatullah *et al*. 2008). Labelling of ICU data can occur manually, automatically or in a semi-automated fashion, but in each case, a standard lexicon is required. There are several standard lexicons for labelling ICU data, depending on the category of data. These include Logical Observation Identifiers, Names and Codes (LOINC; for laboratory and other diagnostic results), the Systematized Nomenclature of Medicine–Clinical Terms (SNOMED-CT; for diseases, findings, procedures, micro-organisms, pharmaceuticals, etc.), Medical Subject Headings (the National Library of Medicine's controlled vocabulary thesaurus of naming descriptors organized in a hierarchical structure) and the International Classification of Diseases (Chen *et al*. 2007).

The Unified Medical Language System (UMLS) acts as an umbrella lexicon for many of these subsystems (although with some enhanced features; Zhang *et al*. 2005). The UMLS is very large and complex, however; it poses significant comprehension problems for users and database maintenance personnel (Gu *et al*. 2000). Furthermore, the UMLS contains omissions of concepts, errors of semantic type classification and concept ambiguities. In particular, there is no one-to-one mapping between sub-lexicons and often multiple UMLS terms are required to describe a particular event or procedure.

We have therefore developed open-source Java software for using a subset of the UMLS to construct descriptors of events in the ICU data (Shu *et al*. 2004). The UMLS descriptors associated with a given event generally consist of an event code (such as ‘C0340535, acute massive pulmonary embolism’) or a *state* code (such as ‘C0018802, congestive heart failure’). The codes are often associated with a qualifier (such as ‘C0184511, improved’, ‘C0205360, stable’ or ‘C0332271, worsening’). Although many of these codes are predefined in drop-down lists to enable rapid and accurate coding, some events necessarily require a new code. In this case, a free text string can be entered by the clinician to see a range of possible codes to select from. We found that spell checking, general abbreviation dictionaries and personalized abbreviation dictionaries were necessary to enable clinicians to find relevant codes in a timely manner.

### (b) Extracting clinical data from text

Much of the data about patients that are not directly measured by computerized instruments is available only in the form of unstructured natural language statements by clinicians. These data can be typed directly by a clinician or transcribed from dictation or handwritten notes. Unfortunately, manual data entry practices and conversion of data into electronic medical records are prone to error. One study (Dean *et al*. 1995) showed that the most common types of data errors in 1995 were omitted and incorrect doses (in UK hospitals) and incorrect and unordered doses (in US hospitals). More recently, Lisby *et al*. (2005) showed that errors in medication ordering and transcription can be frequent and lead to potentially adverse events. However, the most common types of error throughout the medication process were found to be lack of convenient input modalities (forms or entry terminals), unordered drugs, omission of drugs/dosage levels and lack of identity control.

However, inaccurate transcription and data entry is not confined to medications. Recently, we compared manual acceptance of measurements of heart rate and blood pressure from bedside monitors, with measurements gated by robust automatic measures of signal quality (Hug & Clifford 2007). Results showed that the clinically verified BP values exhibit a small but significant bias towards overestimation. In particular, we demonstrated that hypotensive events are often missed by the action of human recording. Other studies (Nelson *et al*. 2005; Vawdrey *et al*. 2007) have also demonstrated the inherent errors in human recording of physiological signals.

Regardless of the method of transcription, the notes must be interpreted by fairly sophisticated algorithms in order to turn them into a structured form that is suitable for searching, modelling and further analysis. We have found that the notes taken by clinicians during the delivery of care are often most difficult to analyse, even when they are typed rather than handwritten. Perhaps owing to the pressing need for speed, these are often poorly organized, full of non-standard abbreviations and typographic errors, and thus pose the greatest challenges to automated processing. By contrast, more formal notes such as discharge summaries, which summarize a patient's hospital stay, are often more carefully written, consciously trying to inform readers other than the writer, and are thus easier to analyse.

We have developed a computer program to extract diseases and procedures attributed to patients in discharge summaries as an aid to semi-automated annotation of our large case collection. The program maps phrases from the text to the approximately 6 million terms that represent approximately 1.5 million concepts listed in the UMLS, and then maps these to the approximately 1 million listed SNOMED-CT concepts. (In practice, of course, very few of these actually appear in clinical texts.) As reported in Long (2007), this program was able to find 93 per cent of the 1326 clinically significant concepts that had been identified in 96 discharge summaries through a manual review by one to three clinicians. However, the program achieves this high level of recall by allowing many irrelevant and misclassified concepts (almost three times as many as the relevant concepts that it finds). Because the purpose of this program was to help annotators find all the relevant concepts, and it is much easier in annotation to reject an unneeded concept than to code a concept de novo, this bias is acceptable for our application. Nevertheless, we would prefer to have an automated annotation tool that finds almost all the needed concepts but few spurious ones.

If we could build a program that ‘understands’ natural language, then it would be relatively easy to pick out the concepts we want to recognize. However, the problem of understanding text is thought to be *artificial intelligence complete*—it would require a program with true human-level intelligence. Of course, we are not anywhere near that in our technical abilities. Therefore, researchers have taken a range of approaches to the extraction problem. At one extreme are systems that use the best available computational linguistics methods to assign likely parts of speech and semantic categories to individual words, to parse the linguistic structure of the phrases, clauses, sentences and paragraphs that express information, and thus to do a deep analysis of the text. Our program takes a more minimalist approach, dividing the text using punctuation, conjunctions, numbers and a few verbs into phrases. Within these, it looks for the maximum length sub-phrase that matches a UMLS concept, without regard to the surrounding text. We have demonstrated that this approach works well when sensitivity is the overriding concern. It needs further enhancement when elimination of irrelevant concepts is important (e.g. when a disease name is mentioned in a note, but is not associated with the patient).

## 5. Summary

Over the last 5 years, we have encountered significant barriers to the analysis of data in the ICU. These include inaccuracies in time stamps, the sparseness or incompleteness of information (such as when databases are not fully integrated, or events are not recorded), non-specific labelling (such as when free text is used instead of a standard medical lexicon), contradictory information (such as when two monitors disagree about a measurement) and simply incorrect information (such as FAs). To some extent, we have addressed many of these issues using data fusion techniques, model construction and automated coding. However, the issues described in this paper still present significant barriers to the use of ICU data for decision support, particularly with respect to the sparseness of the data and the non-specific labelling of clinical information in free text. Despite this, current trends in hospital information systems provide for an optimistic horizon, as increasing volumes of more frequent data are being captured automatically from monitors (together with event codes and signal quality indicators). Hospital information systems are also moving towards using universal lexicons. Furthermore, current trends towards open data storage formats and interchange protocols mean that open source tools we have developed are likely to be generally useful on a wide variety of data.
